# Essential oils mix effect on chicks ileal and caecal microbiota modulation: a metagenomics sequencing approach

**DOI:** 10.3389/fvets.2024.1350151

**Published:** 2024-04-04

**Authors:** Claire Girard, Thibaut Chabrillat, Sylvain Kerros, Philippe Fravalo, Alexandre Thibodeau

**Affiliations:** ^1^Phytosynthese, Mozac, France; ^2^Faculty of Veterinary Medicine, Research Chair in Meat-Safety (CRSV), Université de Montréal, Saint-Hyacinthe, QC, Canada; ^3^Faculty of Veterinary Medicine, Swine and Avian Infectious Disease Research Centre (CRIPA), Université de Montréal, Saint-Hyacinthe, QC, Canada; ^4^Faculty of Veterinary Medicine, Groupe de recherche et d'enseignement en salubrité alimentaire (GRESA), Université de Montréal, Saint-Hyacinthe, QC, Canada

**Keywords:** microbiota modulation, essential oils, *Campylobacter jejuni*, *Salmonella enteritidis*, ileum, caecum, chicken

## Abstract

**Introduction:**

Microbiota plays a pivotal role in promoting the health and wellbeing of poultry. Essential oils (EOs) serve as an alternative solution for modulating poultry microbiota. This study aimed to investigate, using amplicon sequencing, the effect of a complex and well-defined combination of EOs feed supplement on both ileal and caecal broiler microbiota, within the context of *Salmonella* and *Campylobacter* intestinal colonization.

**Material and methods:**

For this experiment, 150-day-old Ross chicks were randomly allocated to two groups: T+ (feed supplementation with EO mix 500 g/t) and T– (non-supplemented). At day 7, 30 birds from each group were orally inoculated with 10^6^ CFU/bird of a *Salmonella enteritidis* and transferred to the second room, forming the following groups: TS+ (30 challenged birds receiving infeed EO mix at 500g/t) and TS– (30 challenged birds receiving a non-supplemented control feed). At day 14, the remaining birds in the first room were orally inoculated with 10^3^ CFU/bird of two strains of *Campylobacter jejuni*, resulting in the formation of groups T+C+ and T–C+. Birds were sacrificed at day 7, D10, D14, D17, and D21. Ileal and caecal microbiota samples were analyzed using Illumina MiSeq sequencing. At D7 and D14, ileal alpha diversity was higher for treated birds (p <0.05).

**Results and discussion:**

No significant differences between groups were observed in caecal alpha diversity (p>0.05). The ileal beta diversity exhibited differences between groups at D7 (*p* < 0.008), D10 (*p* = 0.029), D14 (*p* **=** 0.001) and D17 (*p* **=** 0.018), but not at D21 (*p* = 0.54). For all time points, the analysis indicated that 6 biomarkers were negatively impacted, while 10 biomarkers were positively impacted. *Sellimonas* and *Weissella* returned the lowest (negative) and highest (positive) coefficient, respectively. At each time point, treatments influenced caecal microbiota beta diversity (*p* < 0.001); 31 genera were associated with T+: 10 *Ruminoccocaceae* genera were alternatively more abundant and less abundant from D7, 7 *Lachnospiraceae* genera were alternatively more and less abundant from D10, 6 *Oscillospiraceae* genera were variable depending on the date and 4 *Enterobacteriaceae* differed from D7. During all the experiment, *Campylobacter* decreased in treated birds (*p* < 0.05). This study showed that EO mix modulates ileal and caecal microbiota composition both before and during challenge conditions, increasing alpha diversity, especially in ileum during the early stages of chick life.

## 1 Introduction

Microbiota plays an essential role in the intestinal function, involved in animal welfare and health, contributing to the host's ability to digest nutrients, mount an immune response, regulate behavior, and resist pathogens (1). Research is increasingly focusing on gut microbiota, propelled by advancements in the field of metagenomics and its associated tools. Currently, the manipulation of the microbiota to provide beneficial effects in animal production, especially in broilers, and can be accurately described using sequencing.

The composition and diversity of chicken microbiota are influenced by various parameters, including intestinal section (ileal and caecal), age, environment, breed, diet, and health status (2–5). Numerous instances of dysbiosis that impaired health and productivity of chickens have been reported (6). On farms, the aim is to maintain growth and productivity as well as intestinal health of birds, while dealing with several potential disorders such as behavior, microbiota dysbiosis, and environmental contamination. All these challenges are interconnected. The composition and structure of microbiota are directly linked to these factors as well as growth performance in chickens (2). In recent years, research has been intensifying to understand how to manipulate the chicken microbiota to mitigate problems encountered during chicken rearing.

Non-antibiotics feed additives, such as pre- and probiotics, organics acids, and essential oils (EOs), are currently employed for this purpose, each with different modes of action. Probiotic activities vary depending on the strains used. They can restore or support gut microbial composition (7), modulate gut immune responses (8, 9) and inflammation (10), and produce antimicrobial molecules or postbiotics, thereby enhancing gut health (7, 11). Prebiotics are recognized for their ability to modulate chicken microbiota through mechanisms such as competitive exclusion of pathogens (12), production of antimicrobial factors (13), and stimulation of host immune system (14). Organic acids are mainly known and used for their antibacterial properties, especially against gram-negative bacteria (15). EOs are known for their selective antibacterial properties (16, 17) and have demonstrated beneficial effects on chicken growth, health, and zootechnical performance (18, 19). However, the modes of action of Eos in the gastrointestinal tract are not exhaustively understood. Nevertheless, *in vitro* studies have demonstrated a bacteriostatic activity against *Escherichia coli*, achieved through permeabilization of bacterial cell membranes (20). Other modes of action have been investigated to explain antibacterial properties of EOs, which vary depending on their nature and combination. Inhibition of energy activation via glucose intake (21), leakage of cell components (22–24), loss of cell membrane potential (20, 25, 26), and decreasing effect on internal ATP levels (22, 27) are multiple examples among other EO's proven mode of action. Particularly for *Campylobacter jejuni*, intracellular ATP and potassium contents are affected by EOs, caused by a lack of efflux (28). In the case of *Salmonella*, it has been proved that EOs inhibit the activity of essential enzymes (29).

High-throughput sequencing techniques have been developed to investigate microbiota composition, diversity, and fluctuation. These techniques are abundantly utilized to explore animal microbiota throughout the whole life of the animals (30, 31). These techniques can be possible implementations to monitor microbiota changes that contribute to enhancing poultry production and animal welfare, as well as for controlling foodborne pathogenic bacteria such as *Salmonella* spp. and *Campylobacter* spp. *Salmonella* and *Campylobacter* are leading bacterial causes of human foodborne diseases, with chickens known to be the primary contributors (32–35). These two bacteria can also be part of the chicken intestinal microbiota and, when present in birds, most often do not cause clinical signs (36, 37). Using strict biosecurity measures, cleaning procedures as well as thorough control of all on-farm inputs may yield *Salmonella* and *Campylobacter* free flocks (38), but this remains a challenge for all producers that are actively seeking easier ways to address these troublesome bacteria.

Many studies have focused on the caecal section of chicken's gut microbiota, and few have explored the small intestine. Ileum is known to play a crucial role in intestinal functions such as digestion and nutrient absorption (39, 40). Therefore, it is surprising to find fewer studies on this intestinal part regarding its impact on chicken health. In ileal contents, total bacterial concentrations range from 10^8^ to 10^9^ bacteria per gram (41) with *Lactobacillus* spp. (70%), *Clostridiaceae* (11%), *Streptococcus* spp. (6.5%), and *Enterococcus* spp. (6.5%) being most abundant (42).

This study aimed, therefore, to investigate, using amplicon sequencing, in time, the effect of a complex and well-defined combination of EOs feed supplement on both ileal and caecal chicken microbiota. EOs have already been proven to express an antibacterial activity against foodborne pathogens *in vitro* (29, 43) but diminished effects *in vivo* (44, 45). As *Salmonella* and *Campylobacter* are significant members of the chicken microbiota but may not always be naturally present, we ensured their presence by manually inoculating these foodborne pathogens into the birds. Since the project's aim is to follow the treatment's effect regardless of any other conditions, the inoculation of the pathogens was not considered as a variable in the subsequent analysis.

## 2 Materials and methods

### 2.1 Essential oils mixture composition

Essential oils mixture (EO mix), such as Phyto CSC ^TM^, was provided by Phytosynthese (Mozac, France). A blend of essential oils was incorporated into animal feed, selected *in vitro* its ability to inhibit several pathogens. The composition of ingredients was analyzed in triplicate using a gas chromatograph (GC) Thermo Fisher Trace GC (Thermo Fischer Scientific, Waltham, MA, USA) coupled to a mass spectrometer (MS) Thermo Fisher DSQ I (Thermo Fischer Scientific, Waltham, MA, USA). Carrier gas was helium used at a flow rate of 0.7 ml/min. The column temperature was initially 60°C and then gradually increased at a rate of 5°C/min until it reached 300°C. Diluted samples of 0.5 μl [1:10 (v:v)] were injected. Phyto CSC components were identified by comparing their mass spectra against the NIST 5 mass spectra library (National Institute of Standards and Technology, Gaithersburg, MD, USA).

### 2.2 Animal model

**Figure 1:**
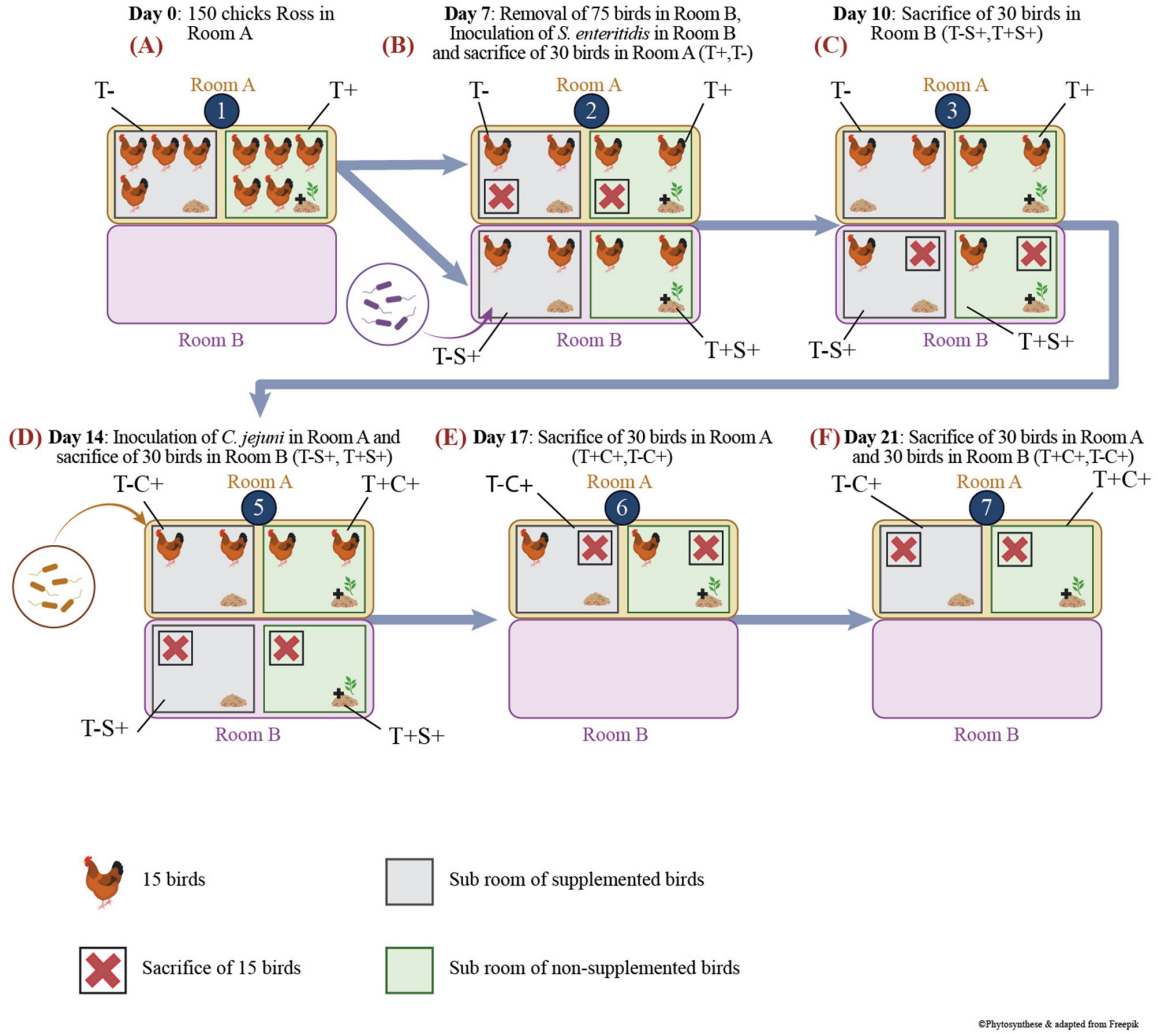
Scheme of experimental protocol and animal model housing. **(A)** Bird's groups and experimentation at D0; **(B)** Bird's groups and experimentation at D7; **(C)** Bird's groups and experimentation at D10; **(D)** Bird's groups and experimentation at D14; **(E)** Bird's groups and experimentation at D17; **(F)** Bird's groups and experimentation at D21.

All experiments on animals were approved by the Comité d'éthique sur l'utilisation des animaux (CEUA) of the veterinary faculty of the Université de Montréal, following guidelines from the Canadian Council on Animal Care (CCAC), project number Rech-1908. Animals were housed at the avian research center of the faculty of veterinary medicine under strict biosecurity (level 2) conditions. All animals had *ad libitum* access to feed and water, and standard in-house lighting and heating procedures were followed. The in-feed treatment immediately started at bird's placement. The experimental scheme is shown in Figure 1. For this experiment, 150-day-old Ross chicks obtained from a local hatchery, vaccinated against Marek disease as well as infectious bronchitis, were randomly allocated to the following 2 groups of 75 birds: T+ (feed supplementation with Phyto CSC 500 g/t) and T– (non-supplemented).

The birds were first housed in a single room, designated as Room A (Figure 1A). At D7, 30 birds from each group were transferred to another room, Room B, within the same facility. Subsequently, the birds were orally inoculated with 1 ml of tryptone-salt (tryptone 0,01% and NaCl 0,085%) containing 10^6^ CFU/bird of a *Salmonella enteritidis* strain, thereby resulting in the following groups: T+S+ (30 challenged birds that received in-feed EO mix at 500 g/t) and T–S+ (30 challenged birds that received a non-supplemented control feed) (Figure 1B).

At D14, remaining (2 × 30) birds in Room A were orally inoculated with 1 ml of tryptone-salt containing two strains of *Campylobacter jejuni* (46), each at 10^3^ CFU/bird, therefore creating groups T+C+ and T–C+ (Figure 1D).

After individual weighing, birds were anesthetized using electronarcosis and euthanized by bleeding. For each necropsy, 15 birds were used per group, and the ileum (last 10 cm before the ileo–caecal junction) and the caecum were removed aseptically and transported to the laboratory on ice. Birds in Room A were sacrificed at day 7 (non-infected: T– and T+) (Figure 1B), D17 (T–C+ and T+C+) (Figure 1E) and D21 (T–C+ and T+C+) (Figure 1F), while birds in Room B were sacrificed at D10 (T–S+ and T+S+) (Figure 1C) and D14 (T–S+ and T+S+) (Figure 1D). In the laboratory, a 1-g sample of caecal and ileal content was placed in a freezing tube and directly plunged in liquid nitrogen to evaluate treatment effects on the microbiota at D7 before the challenges, as well as after 3 and 7 days of *S. enteritidis* and *C. jejuni* challenges (D10, D14, D17, D21). Frozen samples were kept at −80°C until DNA extraction. The weight of sacrificed birds was measured, and the comparison of weight means was analyzed using the Student *t*-test.

### 2.3 Ileal and caecal content and DNA extraction

Total DNA was extracted from −80°C kept sample for each ileal and caecal samples using a combination of a beads-beating lysis and phenol–chloroform purification as previously described (47). A blank sample was extracted alongside the bird samples as a negative control for DNA extraction for downstream use in the molecular biology analysis. DNA concentration was assessed using the Qubit BR assay (Fisher Scientific, Ottawa, ON, Canada) in a DeNovix apparatus (Frogabio, Montréal, Qc, Ca). The DNA samples were diluted to a concentration of 10 ng/μl, aliquoted, and stored at −20°C until further use.

### 2.4 DNA sequencing

Ileal and caecal microbiota samples, as well as DNA extraction negative controls, were analyzed by amplifying and sequencing the V4 region of the 16S rRNA gene from 12 ng of DNA extracted from each bird sample as previously described (46, 48). A positive control, ZymoBIOMICS Microbial Community DNA Standard (Zymo research, Irvine, Ca, USA), and a negative PCR control (water instead of DNA) were also used for quality assessment purpose. The 16S rRNA gene PCR mastermix (25 μl final volume per reaction) consisted of 1x KAPA HiFi HotStart ReadyMix (Kappa Biosystems, Willington, MA, USA), 600 nM of each primer (49), 0.4 mg/ml BSA, and 12.5 ng of DNA. PCR amplifications were confirmed on agarose gels prior to be sent to Genome Québec for Illumina MiSeq 2x250 PE sequencing.

Raw sequences were transferred on Compute Canada Graham server and were first cleaned and denoised using Mothur version 1.44 (50) as recommended by the online MiSeq SOP with the following modifications. For the make.contig command, maxee was set to 2 and deltaq to 5. For the first screen.seq command, maxhomop was set to 70. Sequences were aligned against Silva version 132. Maximum difference for the pre-clustering step was set to 4. Distance.seq cut-off was set at 0.01, and the clustering was done using a cut-off value of 0.005. OTU taxomonic assignation was made using Silva version 138 with the bootstrapping value of 70%. The shared file was created with the label set at 0.005. These parameters differ greatly from what was recommended by the SOP; they had to be developed as the analysis using all settings set at the default values returned no sequence assigned as *Salmonella*, even for those present in the positive mock community control.

### 2.5 Microbiota analysis

The microbiota analysis was conducted with R studio using mainly the phyloseq (51) package and its dependencies. A phyloseq object was firstly created with all samples. Negative and positive controls were inspected. As a quality control step, a NMDS graph was plotted using Bray-Curtis dissimilarity matrix that regrouped all samples. Following quality control, all controls and undesirable samples (too few reads or outliner samples) were removed.

As a first step, for each different site but for all time point, a new phyloseq object was created. For each phyloseq object (Ileum and Caecum), reads were rarefied to the lowest number of sequences within a sample contained in the same phyloseq object. The microbiota was then analyzed for each site of sampling independently. The ADONIS test of the vegan package (52) was run on Bray-Curtis dissimilarities using the “treatment” and “time” as variable. The biomarker analysis at different taxonomic level (MaAsLin2) (53) was also applied to theses samples using the “time” as a random effect and the “treatment” as a fixed effect. As for MaAsLin2 options, no transformation and no normalization were selected, the analysis method was set to “NEGBIN”, the minimal abundance was set at 10, and the minimum prevalence was set at 8 divided by the total of samples analyzed. For all MaAsLin2 analysis, only biomarkers that returned a *p*-value lower than 0.05 and a Q value lower than 0.2 were retained.

Subsequently, for each site, each time point was analyzed separately. For each time point and each sample site, a new phyloseq object was, therefore, created using all samples that passed QC control. Each phyloseq object was rarefied to the lowest number of reads within a sample. Alpha diversity indices (Observed, Shannon and Simpson inverse) were calculated in R Studio, and the treatment effect was assessed using a Wilcoxon test with alpha set at 0.05. The effect of the treatment on the microbiota structure was assessed using the Bray-Curtis dissimilarity index, plotted on NMDS graphs, and analyzed using ADONIS. For the NMDS graph only, the group that received the standard feed, sampled at D7, was kept in the graphical representation for comparison purpose so that the reader may have an idea on the strength of the feed-additive effect. Beta dispersion was also measured using the betadisper function and analyzed using the permutest function with alpha set at 0.05. The biomarker was then identified using MaAsLin2 with options set as just described.

## 3 Results

### 3.1 EO mix composition

The EO mix analysis by GC–MS showed that the commercial preparation contained 68 identifiable components from which the key one was trans-cinnamaldehyde (57.56%), followed by thymol (9.15%) and carvacrol (8.84%) (Table 1).

**Table 1:** Chemical composition of EO mix (gas chromatography–mass spectrometry analysis): 83.5% of components among 68 identified (% area >0.02).

**Components**	**Mean content, %**
Cinnamaldehyde	57.56 ± 0.30
Thymol	9.15 ± 0.10
Carvacrol	8.84 ± 0.09
Eugenol	6.56 ± 0.13
Diallyl disulfide	1.41 ± 0.02

### 3.2 Animal growth

**Figure 2:**
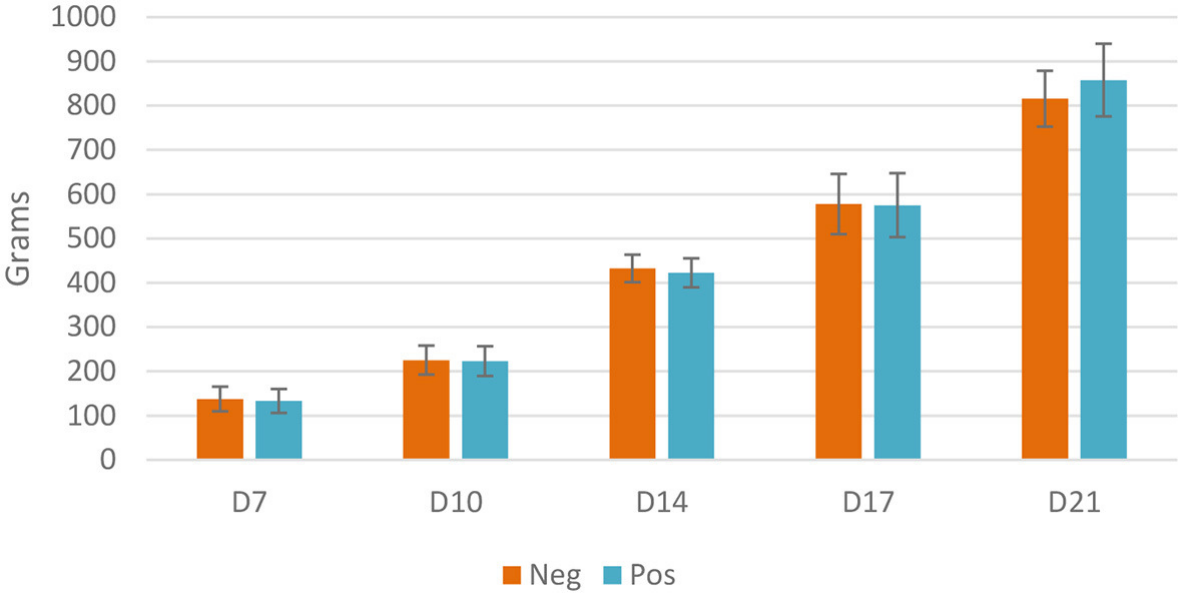
Live weight of euthanized birds, Neg, control group; Pos, EO mix treatment at 500 g/T; no significant difference using the Student *t*-test, *p* < 0.05.

No significant difference on live weight was observed between euthanized birds in different groups; at D21, treated birds tended to reach a higher weight (856g *vs*. 816g, *p* = 0.06). Throughout the experiment, birds achieved expected weight gain, reaching 427 g at D14 and 838 g at D21 (Figure 2).

### 3.3 16s rRNA gene sequencing

Sequencing yielded a total of 37 million sequences. After clean-up, 22 million sequences remained, of which 110,993 were unique. One ileal sample contained only 177 sequences and was immediately removed from the analysis. The PCR-negative controls (only water) contained 13 and 1 sequence, respectively. The controls for DNA extraction contained a mean 36,000 sequences. In the NMDS analysis of all samples, the negative controls were clearly grouped far away from the other samples despite their high sequence counts. Ileal samples consisted of a mean 45,722 sequences (min = 15 257, max = 70 402), while the caecal samples mean sequence count was 96,580 (min = 74 473, max = 123 163). The positive controls (mock community) contained 88,430 and 76,228 sequences, respectively, and matched the expected composition based on the manufacturer recommendation.

As a quality control step, all samples were plotted on a NMDS graph using the Bray-Curtis dissimilarity index to quickly visualize the sample's microbiota and compare it to the controls (Supplementary Figure 1). All sample types (controls and ileal and caecal samples) clustered differently. Only the ileal and caecal samples were retained for the subsequent quality control step. Using the same approach, a quick separate examination was conducted on the microbiota structure of ileal and caecal samples (Supplementary Figures 2, 3). Note that one caecal sample was completely alone and clearly separated from the rest of the data. Therefore, this particular sample was removed from the analysis.

### 3.4 EOs supplementation effect on ileal microbiota

#### 3.4.1 Alpha diversity

**Figure 3:**
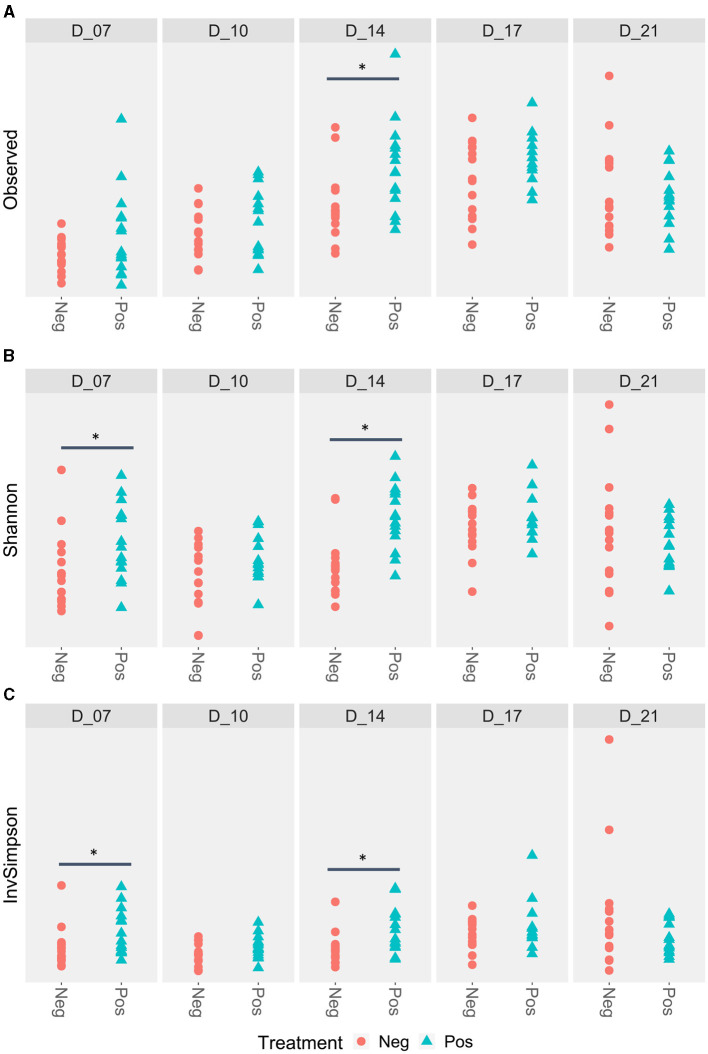
Ileal alpha diversity according to the feed treatment with EO mix (Pos) vs. control group (Neg). *Statistically significant using the Wilcoxon test, *p* < 0.05. **(A)** Ileal alpha diversity according to the Observed index; **(B)** Ileal alpha diversity according to the Shannon index; **(C)** Ileal alpha diversity according to the Simpson inverse index.

The richness of the ileal microbiota of the treated birds was higher at D14. At D7, Shannon and Simpson inverse indexes were also higher for the treated poultry microbiota (Figure 3).

#### 3.4.2 Beta diversity

**Figure 4:**
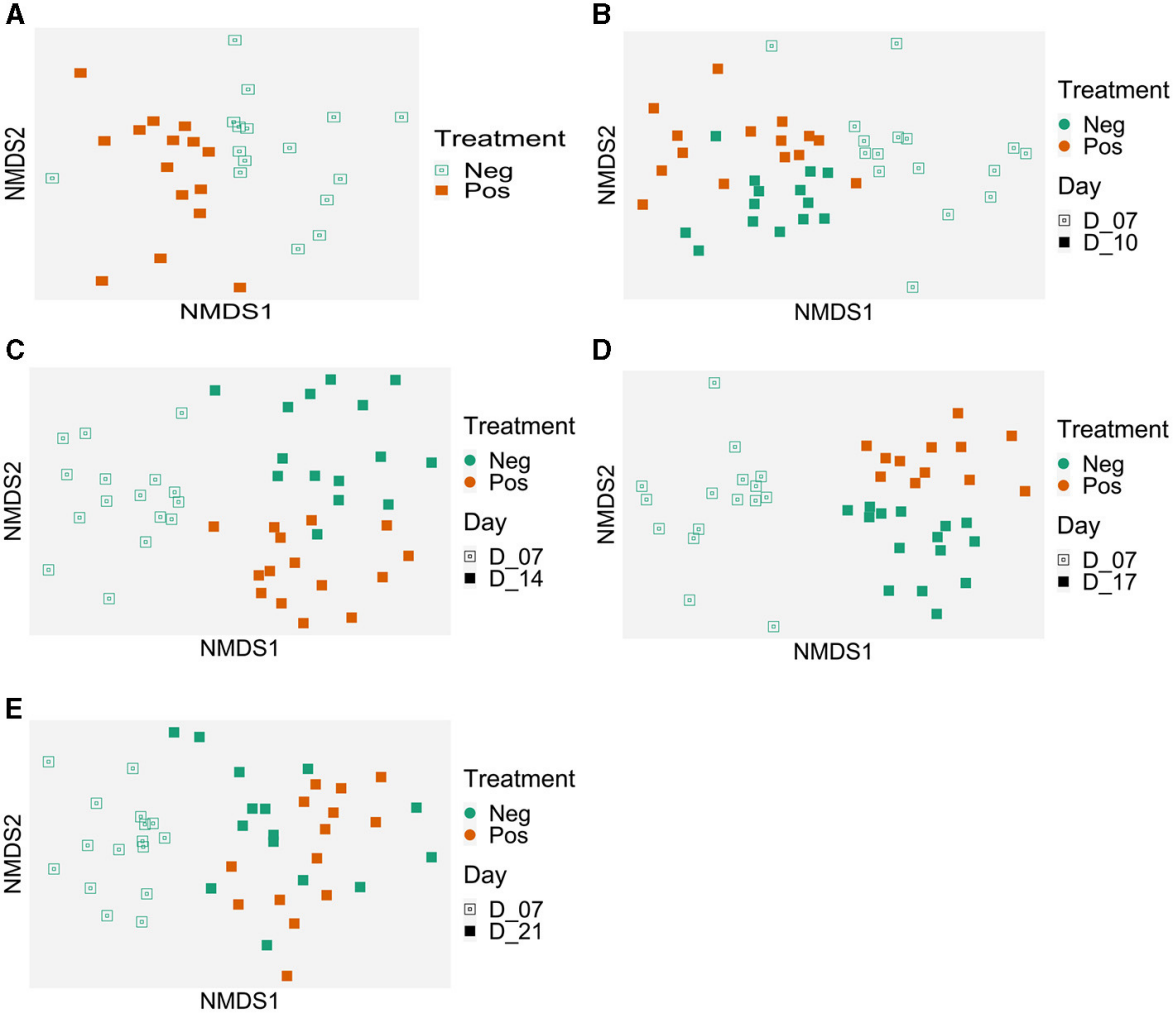
Evolution of the ileal samples microbiota in time compared to the microbiota of 7 days-old control birds. Treatment effect on microbiota structure (Bray-Curtis) represented with Non-metric MultiDimensional Scaling (NMDS) per date: **(A)** 7 days old, *p* = 0.008; **(B)** 10 days old, *p* = 0.029; **(C)** 14 days old, *p* = 0.001; **(D)** 17 days old, *p* = 0.018, and **(E)** 21 days old, *p* = 0.54); beta dispersion *p* > 0.05. Control group (Neg); EO mix supplemented (Pos).

When all ileal samples were analyzed together using the ADONIS test, both time and treatment significantly impacted the microbiota structure and an interaction between the two variables was observed (*p* < 0.001). Therefore, data were analyzed at each time points (Figure 4). In the NMDS analysis, the centroids of each cloud represented by the supplemented and control samples were different at D7 (p <0.008), D10 (*p* = 0.029), D14 (*p* = 0.001), and D17 (*p* = 0.018) but not at D21 (*p* = 0.54) (Figure 4). For the beta dispersion, no significant difference was observed (p>0.05).

Using MaAsLin2, biomarkers were associated with the treatment, using time points as a random effect. Sixteen genera were associated with the treatment (Table 2). *Sellimonas* spp. (coefficient of−2.5, mean of 0.6 sequence per sample for treated birds, mean 6.8 sequence per sample for non-treated birds) and *Weissella* spp. (coefficient of 1.4, mean 343 sequence per sample for treated birds, mean 143 sequences per sample for non-treated birds) returned the lowest (negative) and highest (positive) coefficients (effect size) when comparing animals that received the treatment to those that did not. In addition, for all time points, analysis indicated 6 biomarkers that were negatively impacted (of which 2 *Ruminococcaceae* spp. and 2 *Oscillospiraceae* spp.) and 10 different biomarkers were positively impacted.

**Table 2:** Biomarkers associated with the use of EO mix in the ileum of animals considering all time points.

**Taxonomic assignation**	**Coefficient (treatment *vs*. control, random effect = day of sampling)**	**pval**	**qval**
*Lachnospiraceae.Sellimonas*	−2.5	1.24^E^-12	2.78^E^-11
*Ruminococcaceae.uncultured*	−2.4	6.11^E^-05	3.75^E^-04
*Micrococcaceae.Micrococcaceae_unclassified*	−2.1	4.80^E^-12	7.21^E^-11
*Ruminococcaceae.DTU089*	−2	1.58^E^-03	6.46^E^-03
*Oscillospiraceae.Colidextribacter*	−1.7	2.40^E^-03	9.00^E^-03
*Oscillospiraceae.Flavonifractor*	−1.6	1.42^E^-03	6.41^E^-03
*Lachnospiraceae.Lachnospiraceae_unclassified*	0.4	3.62^E^-02	1.02^E^-01
*Enterobacteriaceae.Escherichia.Shigella*	0.8	3.26^E^-02	9.77^E^-02
*Enterococcaceae.Enterococcus*	0.8	6.67^E^-05	3.75^E^-04
*Eggerthellaceae.Eggerthella*	0.9	1.01^E^-04	5.07^E^-04
*Bacillales_unclassified.Bacillales_unclassified*	0.9	3.22^E^-02	9.77^E^-02
*Corynebacteriaceae.Corynebacterium*	0.9	5.28^E^-06	4.76^E^-05
*Staphylococcaceae.Jeotgalicoccus*	1	4.72^E^-05	3.54^E^-04
*Streptococcaceae.Streptococcus*	1	3.10^E^-06	3.49^E^-05
*Carnobacteriaceae.Jeotgalibaca*	1.3	5.21^E^-03	1.80^E^-02
*Leuconostocaceae.Weissella*	1.4	1.20^E^-15	5.39^E^-14

MaAsLin2 was used for biomarker analysis as described in the material and method section.

The microbiota genus, which were significantly different in the ileum at each time point, is shown in Supplementary Table 1. Eleven genera were only different at one time-point during the trial (for example, *Micrococcaceae.Glutamicibacter* was more abundant at D14 in supplemented group and *Peptostreptococcaceae.Romboutsia* was less represented at D14). Interestingly, *Leuconostocaceae.Weissella* was the lonely genus with a constant and significant effect at all time points. Eight genera were alternatively positive or negative impacted by the treatment (like *Butyricicoccaceae.Butyricicoccus* with a negative coefficient at D7 and positive coefficient at D10 and D14) describing a different reorganization of the microbiota within time.

D14 was the time point with the highest number (22) of bacterial genera impacted by the treatment, in opposition to the others, where only 9 to 12 genus were significantly different.

### 3.5 EOs supplementation effect on caecal microbiota

#### 3.5.1 Alpha diversity

**Figure 5:**
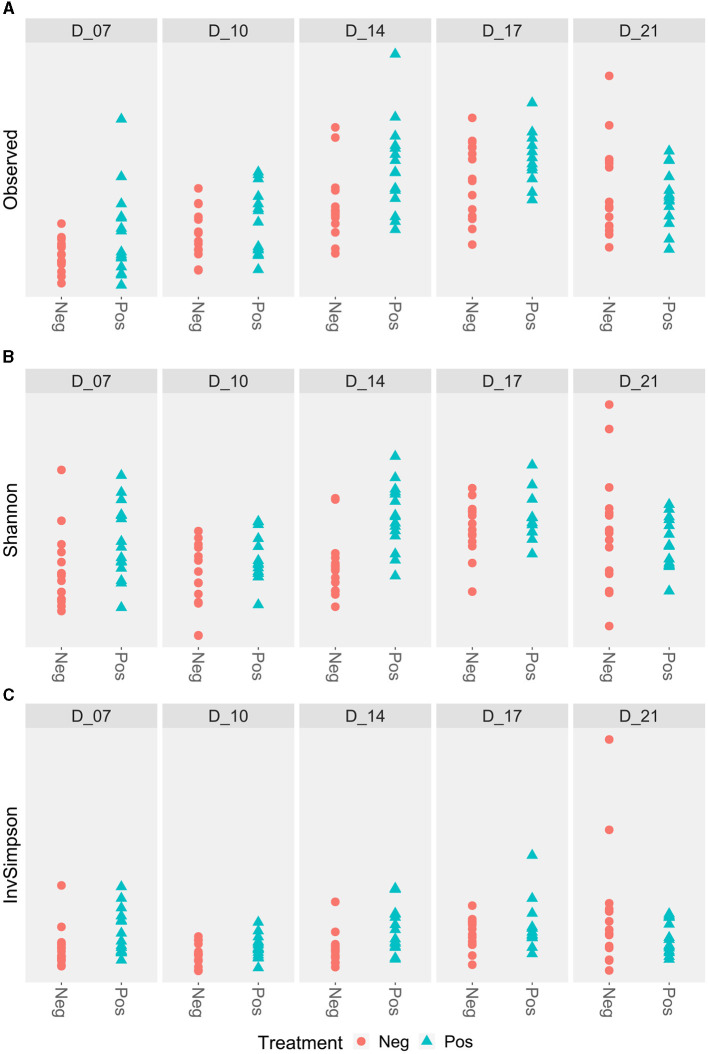
Caecal alpha diversity according to the feed treatment with EO mix (Pos) compared to control group (Neg). *Statistically significant using the Wilcoxon test, *p* < 0.05. No significant difference. **(A)** Caecal alpha diversity according to the Observed index; **(B)** Caecal alpha diversity according to the Shannon index; **(C)** Caecal alpha diversity according to the Simpson inverse index.

No difference was observed for the alpha diversity indexes (Figure 5).

#### 3.5.2 Beta diversity

When all samples were analyzed together, using the ADONIS test, both the variables “Time” and the “Treatment” impacted the microbiota structure (*p* < 0.001). An interaction between the two variables was also observed.

**Figure 6:**
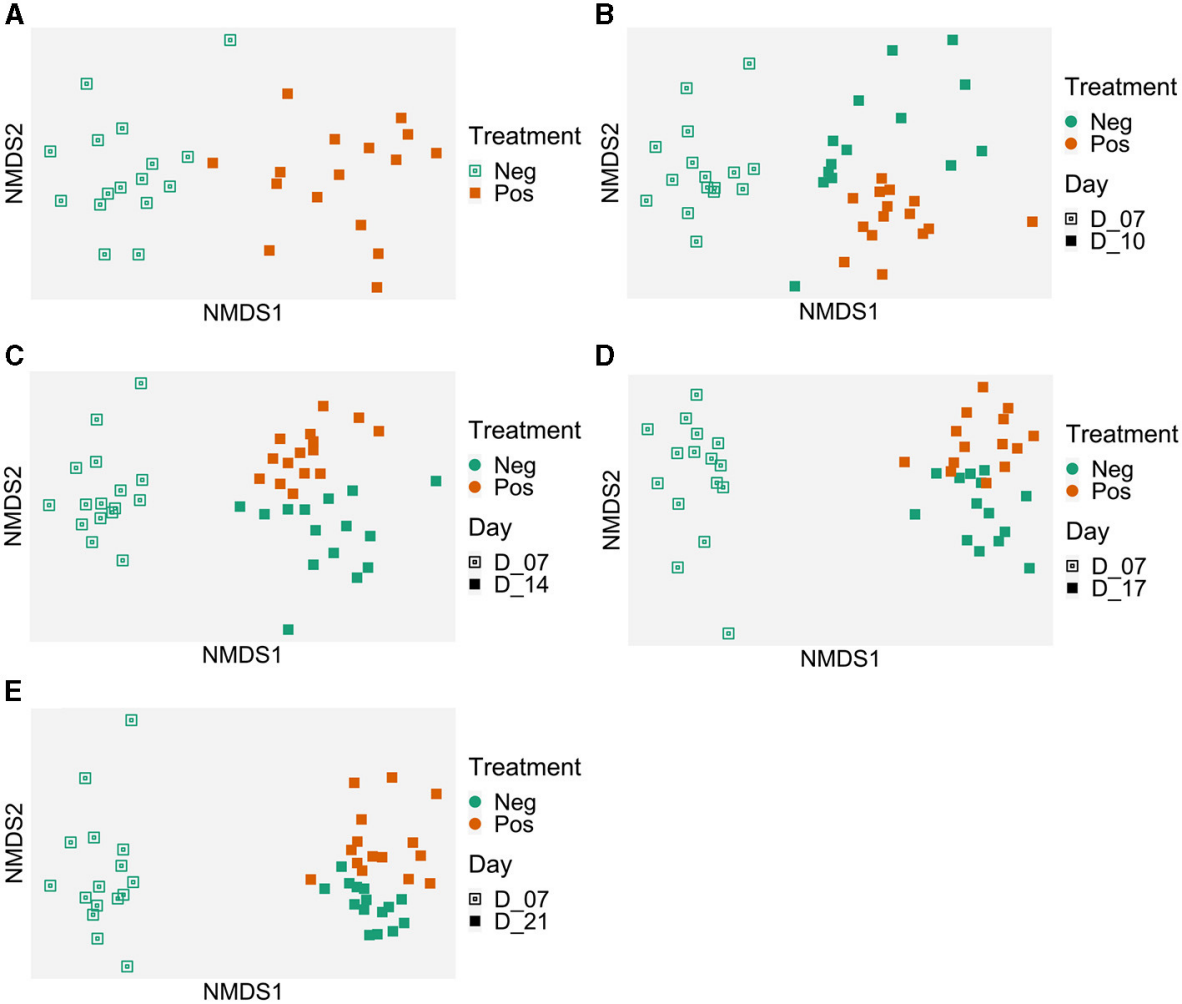
Evolution of the caecal samples microbiota in time compared to the microbiota of 7-day-old control birds. Treatment effect on the microbiota structure (Bray-Curtis) represented with Non-metric MultiDimensional Scaling (NMDS) per date: **(A)** 7 days old, *p* = 0.001; **(B)** 10 days old, *p* = 0.001; **(C)** 14 days old, *p* = 0.001; **(D)** 17 days old, *p* = 0.001; and **(E)** 21 days old, *p* = 0.001. Beta dispersion *p* > 0.05. Control group (Neg); EO mix supplemented (Pos).

Therefore, results were analyzed for each time point. The graphical analysis of caecal beta diversity based on the Bray-Curtis index (Figure 6) demonstrated that bird microbiota of EO mix supplemented group was different from non-supplemented group at each time point: the centroids of points clouds between both groups were different all times of experiment (*p* < 0.001 at D7, D10, D14, D17, and D21). For the beta dispersion, no significant difference was observed (*p* > 0.05). Using MaAsLin2, biomarkers were associated with the treatment using time as a random effect (Table 3, Supplementary Table 2). Thirty-one genera were associated with the treatment. *Erysipelotrichaceae_unclassified* (coefficient of −7, mean 0.04 sequence per sample for treated birds, mean 78 sequences per sample for non-treated birds) and *Merdibacter* (coefficient of 7.5, mean 270 sequences per sample for treated birds, mean 0.14 sequence per sample for non-treated birds) returned the lowest (negative) and highest (positive) coefficients (effect size) when comparing birds that received the treatment to those that did not. Eleven genera were negatively impacted by the treatment and 20 were more abundant. Several caecal microbiota reorganization was noted at each time point (D7, D10, D14, D14, and D17) compared to the control group (Supplementary Table 2): in particular, 10 *Ruminoccocaceae* genera were alternatively more and less abundant from D7, 7 *Lachnospiraceae* genera were alternatively more and less abundant from D10, 6 *Oscillospiraceae* genera were variable depending on the date, and 4 *Enterobacteriaceae* differed from D7. Of note, during all the experiment, *Campylobacteraceae_Campylobacter* decreased in treated birds (*p* < 0.05). Six *Ruminococaceae* taxa and *Leuconostaceae.Weissela* were more abundant in treated group (*p* < 0.05 for all).

**Table 3:** Biomarkers associated with the use of EO mix in the caecum of animals considering all time points.

**Taxonomic Assignation**	**Coefficient (treatment *vs*. control, random effect=day of sampling)**	**pval**	**qval**
*Erysipelotrichaceae.Erysipelotrichaceae_unclassified*	−7.03	2.85E-18	1.88E-16
*Lachnospiraceae.Sellimonas*	−3.86	8.13E-14	1.79E-12
*Lachnospiraceae.GCA.900066575*	−2.56	2.00E-08	1.50E-07
*Campylobacteraceae.Campylobacter*	−2.00	2.93E-02	6.68E-02
*Planococcaceae.Planococcaceae_unclassified*	−1.25	3.63E-02	7.99E-02
*Lachnospiraceae.Eisenbergiella*	−1.01	1.19E-04	4.61E-04
*Lachnospiraceae.Fusicatenibacter*	−0.95	2.00E-02	4.89E-02
*Bacilli_unclassified.Bacilli_unclassified*	−0.72	3.42E-03	1.03E-02
*Oscillospiraceae.Oscillospiraceae_unclassified*	−0.55	3.63E-03	1.04E-02
*Lactobacillales_unclassified.Lactobacillales_unclassified*	−0.31	1.44E-02	3.66E-02
*Lactobacillaceae.Lactobacillus*	−0.24	9.96E-04	3.29E-03
*Lachnospiraceae.Lachnospiraceae_unclassified*	0.24	1.14E-09	1.23E-08
*Lachnospiraceae.Anaerostipes*	0.25	4.81E-02	1.03E-01
*Oscillospiraceae.Flavonifractor*	0.32	1.43E-03	4.49E-03
*Lachnospiraceae.Lachnoclostridium*	0.33	6.17E-06	3.54E-05
*Ruminococcaceae.DTU089*	0.37	2.32E-05	1.10E-04
*Ruminococcaceae.Incertae_Sedis*	0.50	2.05E-08	1.50E-07
*Ruminococcaceae.Caproiciproducens*	0.51	2.03E-05	1.03E-04
*Oscillospirales_fa.Oscillospirales_ge*	0.55	2.15E-02	5.07E-02
*Ruminococcaceae.Ruminococcaceae_unclassified*	0.62	5.21E-16	1.72E-14
*Eggerthellaceae.Eggerthella*	0.92	4.16E-04	1.45E-03
*Corynebacteriaceae.Corynebacterium*	1.11	6.19E-06	3.54E-05
*Staphylococcaceae.Jeotgalicoccus*	1.25	6.43E-06	3.54E-05
*Leuconostocaceae.Weissella*	1.46	1.20E-13	1.99E-12
*Erysipelatoclostridiaceae.Coprobacillus*	1.53	6.40E-03	1.76E-02
*Clostridia_vadinBB60_group_fa.Clostridia_vadinBB60_group_ge*	1.72	1.31E-09	1.23E-08
*Ruminococcaceae.Faecalibacterium*	2.20	7.48E-03	1.98E-02
*Enterobacteriaceae.Salmonella*	3.03	3.04E-05	1.34E-04
*Ruminococcaceae.UBA1819*	3.11	7.14E-05	2.95E-04
*Ruminococcaceae.Subdoligranulum*	3.26	1.34E-04	4.91E-04
*Erysipelotrichaceae.Merdibacter*	7.48	1.26E-10	1.67E-09

MaAsLin2 was used for the biomarker analysis as described in the material and method section.

The 49-microbiota genera, which were significantly different in caecum at different time points, are shown in Supplementary Table 2. Sixteen genera were only different at one time-point during the trial; *Clostridia_vadinBB60_group_fa.Clostridia_vadinBB60_group_ge, Erysipelatoclostridiaceae.Coprobacillus, Lachnospiraceae.Lachnoclostridium, Lachnospiraceae.Lachnospiraceae_unclassified, Leuconostocaceae.Weissella, Ruminococcaceae.DTU089, Ruminococcaceae.Incertae_Sedis* showed a constant positive coefficient at 3 or 4 time points and *Ruminococcaceae.Ruminococcaceae_unclassified* were positively impacted continuously during the trial and *Lachnospiraceae.ASF356, Lachnospiraceae.Eisenbergiella, Lachnospiraceae.GCA.900066575, Lachnospiraceae.Sellimonas, Lactobacillaceae.Lactobacillus* returned a negative coefficient at 3 time points. *Enterobacteriaceae.Salmonella* in caecum was less abundant at D10 and more abundant at D14, D17, and D21 in the treated groups. Similarly, to ileum, 10 genera got both negative and positive coefficients according to different time points. These numerous changes expressed a difference in the organization of the microbiota of birds during the time with or without the supplementation. D14 was the peak of divergence between treated and untreated microbiotas with 31 genera significantly impacted by EO mix followed by D17 with 27 genera.

## 4 Discussion

In this study, the experimental animal model avoided on-farm factors that could have had an effect on digestive microbiota. Consequently, microbiota differences observed in this study were independent of batch effect, experimental building and feed.

In our experimental conditions, ileal alpha diversity was higher in treated birds. Several studies showed that monogastric high diversity microbiota could be more stable or healthier than those with low diversity (54, 55). It is known that the increase in microbiota diversity influences health characteristics, immunity, behaviors, and stress response in chicken (56) and it is also linked to productivity (57). Moreover, in the case of chickens, low FCR is also correlated with high microbiota diversity (58). EO mix supplementation contributed to increase microbiota diversity, which is also reported by Amerah et al. (59) with a cinnamaldehyde and thymol mix.

Chicken supplemented with EO mix had a different microbiota structure compared to non-supplemented chicken. This result is consistent with that of other studies using cinnamaldehyde- or thymol- and carvacrol-based supplementations (60, 61).

*Lachnospiraceae* were significantly higher in ileum of supplemented birds during all the experiment. These bacteria are known to be one of the most important butyrate producers (62). They positively influence gut health performance and anti-inflammatory properties in chicken (63). Interestingly, butyrate preserves intestinal cell barrier in regulating the assembly of tight-junctions (64) and increases the mucin secretion in the small intestine (65). In this study, EO mix supplementation increased *Lachnospiraceae* abundance compared to non-supplemented birds. EO mix is expected to positively modulate the intestinal health.

Three days after *S. enteritidis* challenge, EO mix supplementation exhibited no significant reduction of *Salmonella* genus in the bird's caecal content. This aspect is in contradiction with studies that reported a reduction of *Salmonella* in caecal samples of broilers challenged with *S. enteritidis* at 8 days and supplemented in feed with EO's individual components (66). In their study, birds were supplemented with 0.5% to 0.75% of trans-cinnamaldehyde and 0.75% to 1% of eugenol; these concentrations were much higher in other experiments than those used in this experiment. Carvacrol is reported to be able to accumulate in *Salmonella* cell membrane to alter membrane potential to induce conformational and metabolic change, inhibition of bacterial growth in a dose-dependent manner (67, 68). In our challenged experimental conditions, concentrations of cinnamaldehyde and thymol were possibly not enough to see a direct and significant *Salmonella* reduction in 14 days (Table 2).

EOs are known for their antibacterial activity *in vitro* (69–71). Moreover, EOs show an *in vitro* selective antibacterial activity between pathogenic strains and microbiota beneficial strains (72).

Consequently, EO supplementation had a significant modulation effect on ileal and caecal bacteria genus. One hypothesis in this study is that EO mix could stabilize the ileal microbiota structure due to competitive exclusion. Competitive exclusion is the rapid establishment of a balanced intestinal microbiota by oral route protecting chicken from pathogenic bacteria infection such as *Salmonella* or *Campylobacter* (73). Competitive exclusion was first described by Nurmi and Rantala (74) and is still studied as a control strategy to reduce *Campylobacter* colonization in poultry flocks (75). The precise mechanism of action of this protective effect is unknown because of the complexity of the gut as a habitat for microorganisms and the variety of interactions between bacteria, host, and other microorganisms (76). One of the most likely mode of action could be competition for host mucosa receptor (77). Another possible factor is competition between pathogens and native flora for nutrients (73).

In caecum, EO mix supplementation had an effect on *Campylobacter* reduction 3 days after *C. jejuni* inoculation. This result is consistent with the study of Guyard-Nicodeme et al. (78): the blend of garlic and cinnamon oils resulted in a reduction of 1 log CFU/g in *Campylobacter* caecal count 3 days after inoculation. One mechanism of action could be the EO mix microbiota modulation: microbiota composition was identified by Han et al. (79) as a key element to control pathogenic bacteria such as *C. jejuni* in chickens.

Moreover, during all experiments in this study, *Weissella* genus, belonging to *Leuconostocaceae* family, was a significantly differential bacterial genus in both caecal and ileal microbiota structures of EO mix supplemented chicken compared to the control group. *Weissella* are known to be a part of monogastric microbiota (80). Wang et al. (81) demonstrated a probiotic effect of *Weissella koorensis* in pigs on both daily weight gain and immune response during inflammation. Other *Weissella* species were reported to have a biofilm inhibition effect (82) and *in vitro* anti-inflammatory activity (83).

With the significantly higher proportion of *Weissella* in supplemented chicken, EO mix supplementation may contribute to the development of this beneficial bacterial genus and support the balance of ileal and caecal microbiota. On the other hand, this colonization of beneficial bacteria could be one of the mechanisms of action that led to a significant reduction in *Campylobacter* in caecum 3 days after challenge: the improvement in the growth rate of beneficial bacteria strains in avian gut could be a mode of action explaining *Campylobacter* colonization inhibition as competitive exclusion (84).

As with any scientific studies, limitations are present in the work described. First, culture-based methods could have been employed to monitor *Salmonella* and *Campylobacter* as they offer greater precision than sequencing. Additionally, it would also have been interesting to investigate the impact on birds up to market age. Finally, conduction further research with larger samples is essential to assess any zootechnical impacts that may arise from modifications to the microbiota.

## 5 Conclusion

This study demonstrated that EO mix (Phyto CSC^TM^) modulates the composition of ileal and caecal microbiota before and during challenge conditions with several hypothetic modes of action: competitive exclusion and antibacterial activity. EO mix was considered to have a beneficial effect on the composition of ileal and caecal microbiota in increasing alpha diversity, especially in ileum at the early stages of chick life. If no effect against *Salmonella* was observed in this study, EO mix supplementation had an effect on early *Campylobacter* colonization in the caecum. Although several modifications of bacterial genus were observed, the interpretation of specific functions of these genera inside complex microbiota balance needs further studies for better understanding. DNA sequencing is a helpful technique to describe microbiota modulation in both ileal and caecal sections. Further investigation could be conducted, specifically focusing on the interaction between main bacteria families of the gastrointestinal tracts and the production of metabolites beneficial to the host.

## Data availability statement

The data presented are deposited in the NCBI SRA database, accession number PRJNA1062755 and PRJNA1062768.

## Ethics statement

The animal study was approved by Comité d'éthique sur l'utilisation des animaux (CEUA) of the veterinary faculty of the Université de Montréal. The study was conducted in accordance with the local legislation and institutional requirements.

## Author contributions

CG: Writing – original draft, Methodology, Investigation, Formal analysis, Data curation, Conceptualization. TC: Writing – review & editing, Methodology, Funding acquisition, Formal analysis, Conceptualization. SK: Writing – review & editing, Conceptualization. PF: Writing – review & editing, Project administration, Methodology, Funding acquisition, Conceptualization. AT: Writing – review & editing, Writing – original draft, Supervision, Project administration, Methodology, Investigation, Formal analysis, Data curation, Conceptualization.

## References

[1] Qamar A Waheed J Hamza A Mohyuddin SG Lu Z Namula Z Chen Z Chen JJ. The role of intestinal microbiota in chcken health, intestinal physiology and immunity. J Anim Plant Sci. (2020) 31:221. 10.36899/JAPS.2021.2.0221

[2] Stanley D Hughes RJ Geier MS Moore RJ. Bacteria within the gastrointestinal tract microbiota correlated with improved growth and feed conversion: challenges presented for the identification of performance enhancing probiotic bacteria. Front Microbiol. (2016) 7:187. 10.3389/fmicb.2016.00187 26925052 PMC4760072

[3] Stanley D Geier MS Chen H Hughes RJ Moore RJ. Comparison of fecal and cecal microbiotas reveals qualitative similarities but quantitative differences. BMC Microbiol. (2015) 15:51. 10.1186/s12866-015-0388-6 25887695 PMC4403768

[4] Willson N-L Nattrass GS Hughes RJ Moore RJ Stanley D Hynd PI. Correlations between intestinal innate immune genes and cecal microbiota highlight potential for probiotic development for immune modulation in poultry. Appl Microbiol Biotechnol. (2018) 102:9317–29. 10.1007/s00253-018-9281-1 30151605

[5] Han Z Willer T Pielsticker C Gerzova L Rychlik I Rautenschlein S. Differences in host breed and diet influence colonization by *Campylobacter jejuni* and induction of local immune responses in chicken. Gut Pathog. (2016) 8:56. 10.1186/s13099-016-0133-1 27843492 PMC5105272

[6] Wilcox CH Sandilands V Mayasari N Asmara IY Anang A. A literature review of broiler chicken welfare, husbandry, and assessment. Worlds Poult Sci J. (2023) 80:1–30. 10.1080/00439339.2023.2264824

[7] Kulkarni RR Gaghan C Gorrell K Sharif S Taha-Abdelaziz K. Probiotics as alternatives to antibiotics for the prevention and control of necrotic enteritis in chickens. Pathogens. (2022) 11:692. 10.3390/pathogens11060692 35745546 PMC9229159

[8] Brisbin JT Gong J Orouji S Esufali J Mallick AI Parvizi P. Oral Treatment of chickens with *Lactobacilli* influences elicitation of immune responses. Clin Vaccine Immunol. (2011) 18:1447–55. 10.1128/CVI.05100-11 21734067 PMC3165221

[9] Alizadeh M Shojadoost B Astill J Taha-Abdelaziz K Karimi SH Bavananthasivam J. Effects of in ovo inoculation of multi-strain *Lactobacilli* on cytokine gene expression and antibody-mediated immune responses in chickens. Front Vet Sci. (2020) 7:105. 10.3389/fvets.2020.00105 32185187 PMC7058628

[10] Peng X Ed-Dra A Song Y Elbediwi M Nambiar RB Zhou X. Lacticaseibacillus rhamnosus alleviates intestinal inflammation and promotes microbiota-mediated protection against *Salmonella* fatal infections. Front Immunol. (2022) 13:973224. 10.3389/fimmu.2022.973224 36032095 PMC9411107

[11] Johnson CN Kogut MH Genovese K He H Kazemi S Arsenault RJ. Administration of a postbiotic causes immunomodulatory responses in broiler gut and reduces disease pathogenesis following challenge. Microorganisms. (2019) 7:268. 10.3390/microorganisms7080268 31426502 PMC6723925

[12] Callaway TR Edrington TS Anderson RC Harvey RB Genovese KJ Kennedy CN. Probiotics, prebiotics and competitive exclusion for prophylaxis against bacterial disease. Anim Health Res Rev. (2008) 9:217–25. 10.1017/S1466252308001540 19102792

[13] Chen Y-S Srionnual S Onda T Yanagida F. Effects of prebiotic oligosaccharides and trehalose on growth and production of bacteriocins by lactic acid bacteria. Lett Appl Microbiol. (2007) 45:190–3. 10.1111/j.1472-765X.2007.02167.x 17651217

[14] Mohd Shaufi MA Sieo CC Chong CW Gan HM Ho YW. Deciphering chicken gut microbial dynamics based on high-throughput 16S rRNA metagenomics analyses. Gut Pathog. (2015) 7:4. 10.1186/s13099-015-0051-7 25806087 PMC4372169

[15] Adamczak A Ożarowski M Karpiński TM. Antibacterial activity of some flavonoids and organic acids widely distributed in plants. J Clin Med. (2019) 9:109. 10.3390/jcm9010109 31906141 PMC7019947

[16] Ambrosio CMS de Alencar SM de Sousa RLM Moreno AM Da Gloria EM. Antimicrobial activity of several essential oils on pathogenic and beneficial bacteria. Ind Crops Prod. (2017) 97:128–36. 10.1016/j.indcrop.2016.11.045

[17] Ambrosio CMS Ikeda NY Miano AC Saldaña E Moreno AM Stashenko E. Unraveling the selective antibacterial activity and chemical composition of citrus essential oils. Sci Rep. (2019) 9:17719. 10.1038/s41598-019-54084-3 31776388 PMC6881395

[18] Weber GM Michalczuk M Huyghebaert G Juin H Kwakernaak C Gracia MI. Effects of a blend of essential oil compounds and benzoic acid on performance of broiler chickens as revealed by a meta-analysis of 4 growth trials in various locations. Poult Sci. (2012) 91:2820–8. 10.3382/ps.2012-02243 23091138

[19] Bozkurt M Küçükyilmaz K Pamukçu M çabuk M Alçiçek A çatli AU. Long-term effects of dietary supplementation with an essential oil mixture on the growth and laying performance of two layer strains. Ital J Anim Sci. (2012) 11:e5. 10.4081/ijas.2012.e5

[20] Girard C Fayolle K Kerros S Leriche F. Flow cytometric assessment of the antimicrobial properties of an essential oil mixture against *Escherichia coli*. J Anim Feed Sci. (2019) 28:187–98. 10.22358/jafs/109687/2019

[21] Gill AO Holley RA. Mechanisms of Bactericidal Action of Cinnamaldehyde against *Listeria monocytogenes* and of Eugenol against *L. monocytogenes* and Lactobacillus sakei. Appl Environ Microbiol. (2004) 70:5750–5. 10.1128/AEM.70.10.5750-5755.2004 15466510 PMC522076

[22] Helander IM Alakomi H-L Latva-Kala K Mattila-Sandholm T Pol I Smid EJ. Characterization of the action of selected essential oil components on gram-negative bacteria. J Agric Food Chem. (1998) 46:3590–5. 10.1021/jf980154m

[23] Xu J Zhou F Ji B-P Pei R-S Xu N. The antibacterial mechanism of carvacrol and thymol against *Escherichia coli*. Lett Appl Microbiol. (2008) 47:174–9. 10.1111/j.1472-765X.2008.02407.x 19552781

[24] Carson CF Mee BJ Riley TV. Mechanism of action of *Melaleuca alternifolia* (tea tree) oil on *Staphylococcus aureus* Determined by time-kill, lysis, leakage, and salt tolerance assays and electron microscopy. Antimicrob Agents Chemother. (2002) 46:1914–20. 10.1128/AAC.46.6.1914-1920.2002 12019108 PMC127210

[25] Fisher K Phillips C. The mechanism of action of a citrus oil blend against *Enterococcus faecium* and *Enterococcus faecalis*. J Appl Microbiol. (2009) 106:1343–9. 10.1111/j.1365-2672.2008.04102.x 19187138

[26] Ultee A Bennik MHJ Moezelaar R. The phenolic hydroxyl group of carvacrol is essential for action against the food-borne pathogen *Bacillus cereus*. Appl Environ Microbiol. (2002) 68:1561–8. 10.1128/AEM.68.4.1561-1568.2002 11916669 PMC123826

[27] Cox S Mann C Markham J Gustafson J Warmington J Wyllie S. Determining the antimicrobial actions of tea tree oil. Molecules. (2001) 6:87–91. 10.3390/60100087

[28] Dedieu L Brunel JM Lorenzi V Muselli A Berti L Bolla JM. Antibacterial mode of action of the *Daucus carota* essential oil active compounds against *Campylobacter jejuni* and efflux-mediated drug resistance in gram-negative bacteria. Molecules. (2020) 25:5448. 10.3390/molecules25225448 33233754 PMC7699865

[29] Ebani VV Nardoni S Bertelloni F Tosi G Massi P Pistelli L. *In vitro* antimicrobial activity of essential oils against *Salmonella enterica* Serotypes *Enteritidis* and *Typhimurium* strains isolated from poultry. Molecules. (2019) 24:900. 10.3390/molecules24050900 30836721 PMC6429372

[30] Feye KM Thompson DR Rothrock MJ Kogut MH Ricke SC. Poultry processing and the application of microbiome mapping. Poult Sci. (2020) 99:678–88. 10.1016/j.psj.2019.12.019 32029154 PMC7587767

[31] Haynes E Jimenez E Pardo MA Helyar SJ. The future of NGS (Next Generation Sequencing) analysis in testing food authenticity. Food Control. (2019) 101:134–43. 10.1016/j.foodcont.2019.02.010

[32] Hermans D Pasmans F Messens W Martel A Van Immerseel F Rasschaert G. Poultry as a host for the zoonotic pathogen *Campylobacter jejuni*. Vector-Borne Zoonotic Dis. (2012) 12:89–98. 10.1089/vbz.2011.0676 22133236

[33] Bajpai VK Baek K-H Kang SC. Control of *Salmonella* in foods by using essential oils: a review. Food Res Int. (2012) 45:722–34. 10.1016/j.foodres.2011.04.052

[34] Poppe C. Salmonella enteritidis in Canada. Int J Food Microbiol. (1994) 21:1–5. 10.1016/0168-1605(94)90193-7 8155467

[35] Fantasia M Filetici E. Salmonella enteritidis in Italy. Int J Food Microbiol. (1994) 21:7–13. 10.1016/0168-1605(94)90194-5 8155480

[36] Rahimi E Alipoor-Amroabadi M Khamesipour F. Investigation of prevalence of thermotolerant *Campylobacter* spp. in livestock feces. Can J Anim Sci. (2017) 97:207–13. 10.1139/cjas-2015-0166

[37] Rukambile E Sintchenko V Muscatello G Kock R Alders R. Infection, colonization and shedding of *Campylobacter* and *Salmonella* in animals and their contribution to human disease: a review. Zoonoses Public Health. (2019) 66:562–78. 10.1111/zph.12611 31179637

[38] Fraser RW Williams NT Powell LF Cook AJC. Reducing *Campylobacter* and *Salmonella* infection: two studies of the economic cost and attitude to adoption of on-farm biosecurity measures: farmer attitudes and control of foodborne zoonoses. Zoonoses Public Health. (2010) 57:e109–15. 10.1111/j.1863-2378.2009.01295.x 19968845

[39] Hübener K Vahjen W Simon O. Bacterial responses to different dietary cereal types and xylanase supplementation in the intestine of broiler chicken. Arch Für Tierernaehrung. (2002) 56:167–87. 10.1080/00039420214191 12391903

[40] Gong J Forster RJ Yu H Chambers JR Wheatcroft R Sabour PM Chen S. Molecular analysis of bacterial populations in the ileum of broiler chickens and comparison with bacteria in the cecum. FEMS Microbiol Ecol. (2002) 41:171–9. 10.1111/j.1574-6941.2002.tb00978.x 19709251

[41] Apajalahti J Kettunen A Graham H. Characteristics of the gastrointestinal microbial communities, with special reference to the chicken. Worlds Poult Sci J. (2004) 60:223–32. 10.1079/WPS20040017

[42] Lu J Idris U Harmon B Hofacre C Maurer JJ Lee MD. Diversity and succession of the intestinal bacterial community of the maturing broiler chicken. Appl Environ Microbiol. (2003) 69:6816–24. 10.1128/AEM.69.11.6816-6824.2003 14602645 PMC262306

[43] Mutlu-ingok A Firtin B Karbancioglu-guler F. Chemical composition and comparative antibacterial properties of basil essential oil against clinical and standard strains of campylobacter spp ACTA. Pharm Sci. (2019) 57:183. 10.23893/1307-2080.APS.05711

[44] Lang M Montjarret A Duteil E Bedoux G. Cinnamomum cassia and *Syzygium aromaticum* essential oils reduce the colonization of *Salmonella typhimurium* in an *in vivo* infection model using *Caenorhabditis elegans. Molecules*. (2021) 26:5598. 10.3390/molecules26185598 PMC8467367 34577068

[45] Szott V Reichelt B Alter T Friese A Roesler U. *In vivo* efficacy of carvacrol on *Campylobacter jejuni* prevalence in broiler chickens during an entire fattening period. Eur J Microbiol Immunol. (2020) 10:131–8. 10.1556/1886.2020.00011 32750025 PMC7592510

[46] Chagneau S Gaucher M-L Thériault WP Fravalo P Thibodeau A. Observations supporting hypothetical commensalism and competition between two *Campylobacter jejuni* strains colonizing the broiler chicken gut. Front Microbiol. (2023) 13:1071175. 10.3389/fmicb.2022.1071175 36817113 PMC9937062

[47] Thibodeau A Fravalo P Yergeau E Arsenault J Lahaye L Letellier A. Chicken caecal microbiome modifications induced by Campylobacter jejuni colonization and by a non-antibiotic feed additive. PLoS ONE. (2015) 10:e0131978. 10.1371/journal.pone.0131978 26161743 PMC4498643

[48] Trudeau S Thibodeau A Côté J-C Gaucher M-L Fravalo P. Contribution of the broiler breeders' fecal microbiota to the establishment of the eggshell microbiota. Front Microbiol. (2020) 11:666. 10.3389/fmicb.2020.00666 32351488 PMC7176364

[49] Caporaso JG Lauber CL Walters WA Berg-Lyons D Huntley J Fierer N. Ultra-high-throughput microbial community analysis on the Illumina HiSeq and MiSeq platforms. ISME J. (2012) 6:1621–4. 10.1038/ismej.2012.8 22402401 PMC3400413

[50] Schloss PD Westcott SL Ryabin T Hall JR Hartmann M Hollister EB. Introducing mothur: open-source, platform-independent, community-supported software for describing and comparing microbial communities. Appl Environ Microbiol. (2009) 75:7537–41. 10.1128/AEM.01541-09 19801464 PMC2786419

[51] McMurdie PJ Holmes S. phyloseq: An R package for reproducible interactive analysis and graphics of microbiome census data. PLoS ONE. (2013) 8:e61217. 10.1371/journal.pone.0061217 23630581 PMC3632530

[52] Oksanen J Blanchet G Kindt R Legendre P Minchin PR O'Hara RB. Vegan: Community Ecology Package. (2015). Available online at: http://CRAN.R-project.org/package=vegan (accessed June 1, 2023).

[53] Mallick H Rahnavard A McIver LJ Ma S Zhang Y Nguyen LH. Multivariable association discovery in population-scale meta-omics studies. PLOS Comput Biol. (2021) 17:e1009442. 10.1371/journal.pcbi.1009442 34784344 PMC8714082

[54] Lozupone CA Stombaugh JI Gordon JI Jansson JK Knight R. Diversity, stability and resilience of the human gut microbiota. Nature. (2012) 489:220–30. 10.1038/nature11550 22972295 PMC3577372

[55] Clemente JC Ursell LK Parfrey LW Knight R. The impact of the gut microbiota on human health: an integrative view. Cell. (2012) 148:1258–70. 10.1016/j.cell.2012.01.035 22424233 PMC5050011

[56] Chen S Luo S Yan C. Gut microbiota implications for health and welfare in farm animals: a review. Animals. (2021) 12:93. 10.3390/ani12010093 35011199 PMC8749645

[57] Kraimi N Dawkins M Gebhardt-Henrich SG Velge P Rychlik I Volf J. Influence of the microbiota-gut-brain axis on behavior and welfare in farm animals: a review. Physiol Behav. (2019) 210:112658. 10.1016/j.physbeh.2019.112658 31430443

[58] Diaz Carrasco JM Casanova NA Fernández Miyakawa ME. Microbiota, gut health and chicken productivity: what is the connection? Microorganisms. (2019) 7:374. 10.3390/microorganisms7100374 31547108 PMC6843312

[59] Amerah AM Péron A Zaefarian F Ravindran V. Influence of whole wheat inclusion and a blend of essential oils on the performance, nutrient utilisation, digestive tract development and ileal microbiota profile of broiler chickens. Br Poult Sci. (2011) 52:124–32. 10.1080/00071668.2010.548791 21337207

[60] Chen Y Wang J Yu L Xu T Zhu N. Microbiota and metabolome responses in the cecum and serum of broiler chickens fed with plant essential oils or virginiamycin. Sci Rep. (2020) 10:5382. 10.1038/s41598-020-60135-x 32214106 PMC7096418

[61] Pham VH Kan L Huang J Geng Y Zhen W Guo Y. Dietary encapsulated essential oils and organic acids mixture improves gut health in broiler chickens challenged with necrotic enteritis. J Anim Sci Biotechnol. (2020) 11:18. 10.1186/s40104-019-0421-y 32110391 PMC7033934

[62] Pryde SE Duncan SH Hold GL Stewart CS Flint HJ. The microbiology of butyrate formation in the human colon. FEMS Microbiol Lett. (2002) 217:133–9. 10.1111/j.1574-6968.2002.tb11467.x 12480096

[63] Onrust L Ducatelle R Van Driessche K De Maesschalck C Vermeulen K Haesebrouck F. Steering endogenous butyrate production in the intestinal tract of broilers as a tool to improve gut health. Front Vet Sci. (2015) 2:75. 10.3389/fvets.2015.00075 26734618 PMC4682374

[64] Peng L Li Z-R Green RS Holzman IR Lin J. Butyrate enhances the intestinal barrier by facilitating tight junction assembly via activation of AMP-activated protein kinase in caco-2 cell monolayers. J Nutr. (2009) 139:1619–25. 10.3945/jn.109.104638 19625695 PMC2728689

[65] Smirnov A Perez R Amit-Romach E Sklan D Uni Z. Mucin dynamics and microbial populations in chicken small intestine are changed by dietary probiotic and antibiotic growth promoter supplementation. J Nutr. (2005) 135:187–92. 10.1093/jn/135.2.187 15671211

[66] Kollanoor-Johny A Mattson T Baskaran SA Amalaradjou MA Babapoor S March B. Reduction of *Salmonella enterica* Serovar *Enteritidis* colonization in 20-day-old broiler chickens by the plant-derived compounds *trans*-Cinnamaldehyde and Eugenol. Appl Environ Microbiol. (2012) 78:2981–7. 10.1128/AEM.07643-11 22327574 PMC3318785

[67] Rodriguez-Garcia I Silva-Espinoza BA Ortega-Ramirez LA Leyva JM Siddiqui MW Cruz-Valenzuela MR. Oregano essential oil as an antimicrobial and antioxidant additive in food products. Crit Rev Food Sci Nutr. (2016) 56:1717–27. 10.1080/10408398.2013.800832 25763467

[68] Di Vito M Cacaci M Barbanti L Martini C Sanguinetti M Benvenuti S. Origanum vulgare essential oil *vs*. a commercial mixture of essential oils: in vitro effectiveness on salmonella spp from poultry and swine intensive livestock. Antibiotics. (2020) 9:763. 10.3390/antibiotics9110763 33142685 PMC7693145

[69] Mickiene R Bakutis B Baliukoniene V. Antimicrobial activity of two essential oils. Ann Agric Environ Med. (2011) 8:139–44. 21739934

[70] Liu Y Zhang X Wang Y Chen F Yu Z Wang L. Effect of citrus lemon oil on growth and adherence of *Streptococcus mutans*. World J Microbiol Biotechnol. (2013) 29:1161–7. 10.1007/s11274-013-1275-7 23381618

[71] Bassolé IHN Juliani HR. Essential oils in combination and their antimicrobial properties. Molecules. (2012) 17:3989–4006. 10.3390/molecules17043989 22469594 PMC6268925

[72] Ouwehand AC Tiihonen K Kettunen H Peuranen S Schulze H Rautonen N. In vitro effects of essential oils on potential pathogens and beneficial members of the normal microbiota. Vet Med (Praha). (2010) 55:71–8. 10.17221/152/2009-VETMED

[73] Mead GC. Prospects for ‘competitive exclusion' treatment to control *salmonellas* and other foodborne pathogens in poultry. Vet J. (2000) 159:111–23. 10.1053/tvjl.1999.0423 10712799

[74] Nurmi E Rantala M. New aspects of *Salmonella* infection in broiler production. Nature. (1973) 241:210–1. 10.1038/241210a0 4700893

[75] Szott V Reichelt B Friese A Roesler U. A complex competitive exclusion culture reduces *Campylobacter jejuni* colonization in broiler chickens at slaughter age *in vivo*. Vet Sci. (2022) 9:181. 10.3390/vetsci9040181 35448680 PMC9029414

[76] Rolfe RD. Population dynamics of the intestinal tract. In: Colon Control Hum Bact Enteropathologens Poult. San Diego, CA: Academic Press (1991).

[77] Snoeyenbos GH Weinack OM Smyser CF. Further studies on competitive exclusion for controlling *Salmonellae* in chickens. Avian Dis. (1979) 23:904. 10.2307/1589607 546412

[78] Guyard-Nicodème M Rivoal K Houard E Rose V Quesne S Mourand G. Prevalence and characterization of *Campylobacter jejuni* from chicken meat sold in French retail outlets. Int J Food Microbiol. (2015) 203:8–14. 10.1016/j.ijfoodmicro.2015.02.013 25770428

[79] Han Z Pielsticker C Gerzova L Rychlik I Rautenschlein S. The influence of age on *Campylobacter jejuni* infection in chicken. Dev Comp Immunol. (2016) 62:58–71. 10.1016/j.dci.2016.04.020 27131855

[80] Fusco V. The genus *Weissella*: taxonomy, ecology and biotechnological potential. Front Microbiol. (2015) 6:22. 10.3389/fmicb.2015.00155 25852652 PMC4362408

[81] Wang JP Yoo JS Jang HD Lee JH Cho JH Kim IH. Effect of dietary fermented garlic by *Weissella koreensis* powder on growth performance, blood characteristics, and immune response of growing pigs challenged with Escherichia coli lipopolysaccharide. J Anim Sci. (2011) 89:2123–31. 10.2527/jas.2010-3186 21317348

[82] Kang M-S Chung J Kim S-M Yang K-H Oh J-S. Effect of *Weissella cibaria* Isolates on the Formation of *Streptococcus mutans* Biofilm. Caries Res. (2006) 40:418–425. 10.1159/000094288 16946611

[83] Kang M-S Lim H-S Kim S-M Lee H-C Oh J-S. Effect of *Weissella cibaria* on *Fusobacterium nucleatum*-induced interleukin-6 and interleukin-8 production in KB cells. J Bacteriol Virol. (2011) 41:9. 10.4167/jbv.2011.41.1.9

[84] Santini C Baffoni L Gaggia F Granata M Gasbarri R Di Gioia D. Characterization of probiotic strains: an application as feed additives in poultry against Campylobacter jejuni. Int J Food Microbiol. (2010) 141:S98–S108. 10.1016/j.ijfoodmicro.2010.03.039 20452074

