# Supplementary material for: Essential oils mix effect on chicks ileal and caecal microbiota modulation: a metagenomics sequencing approach
Source: Front Vet Sci. 2024 Apr 4;11:1350151. doi: 10.3389/fvets.2024.1350151 (PMC11025455; doi:10.3389/fvets.2024.1350151)
Supplement: Supplementary file 1 [file Data_Sheet_1.docx]

Supplementary Material


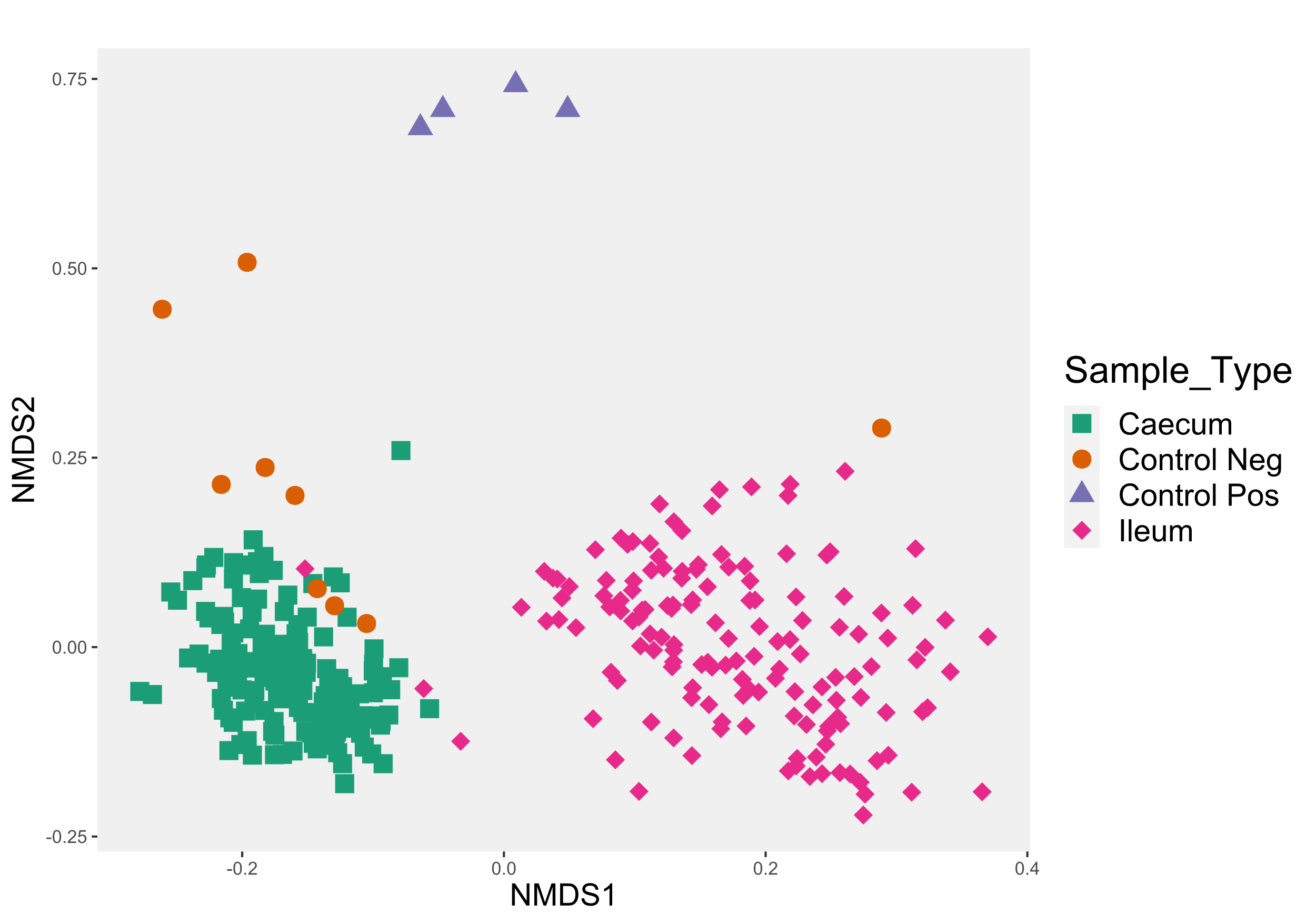


**Figure S1: Graphic** **represented with Non-metric MultiDimensional Scaling (NMDS) of all microbiota sequenced samples (Bray-Curtis)**


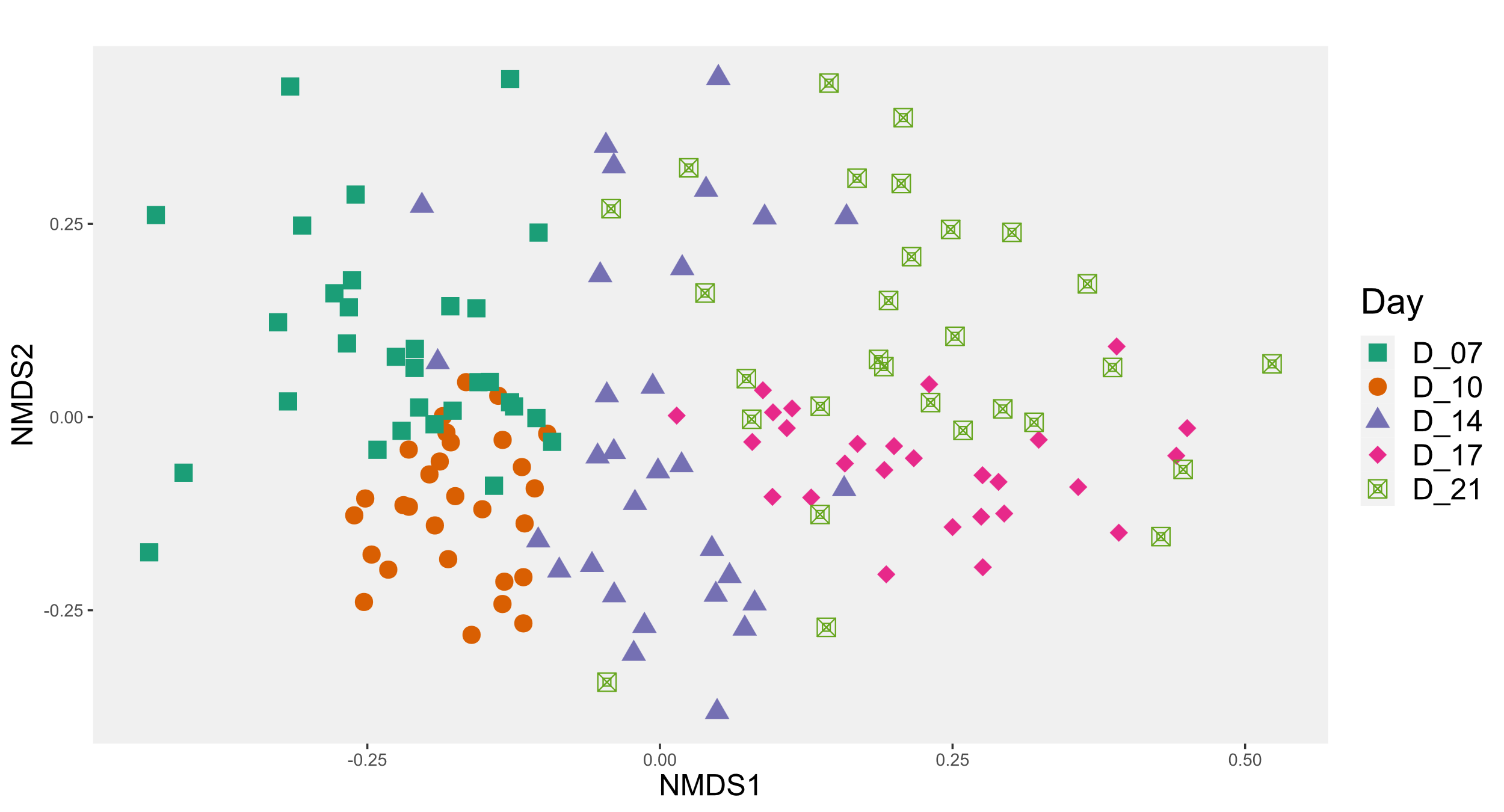


**Figure S2: Graphic represented with Non-metric MultiDimensional Scaling (NMDS) of all ileal sequenced samples (Bray-Curtis)**


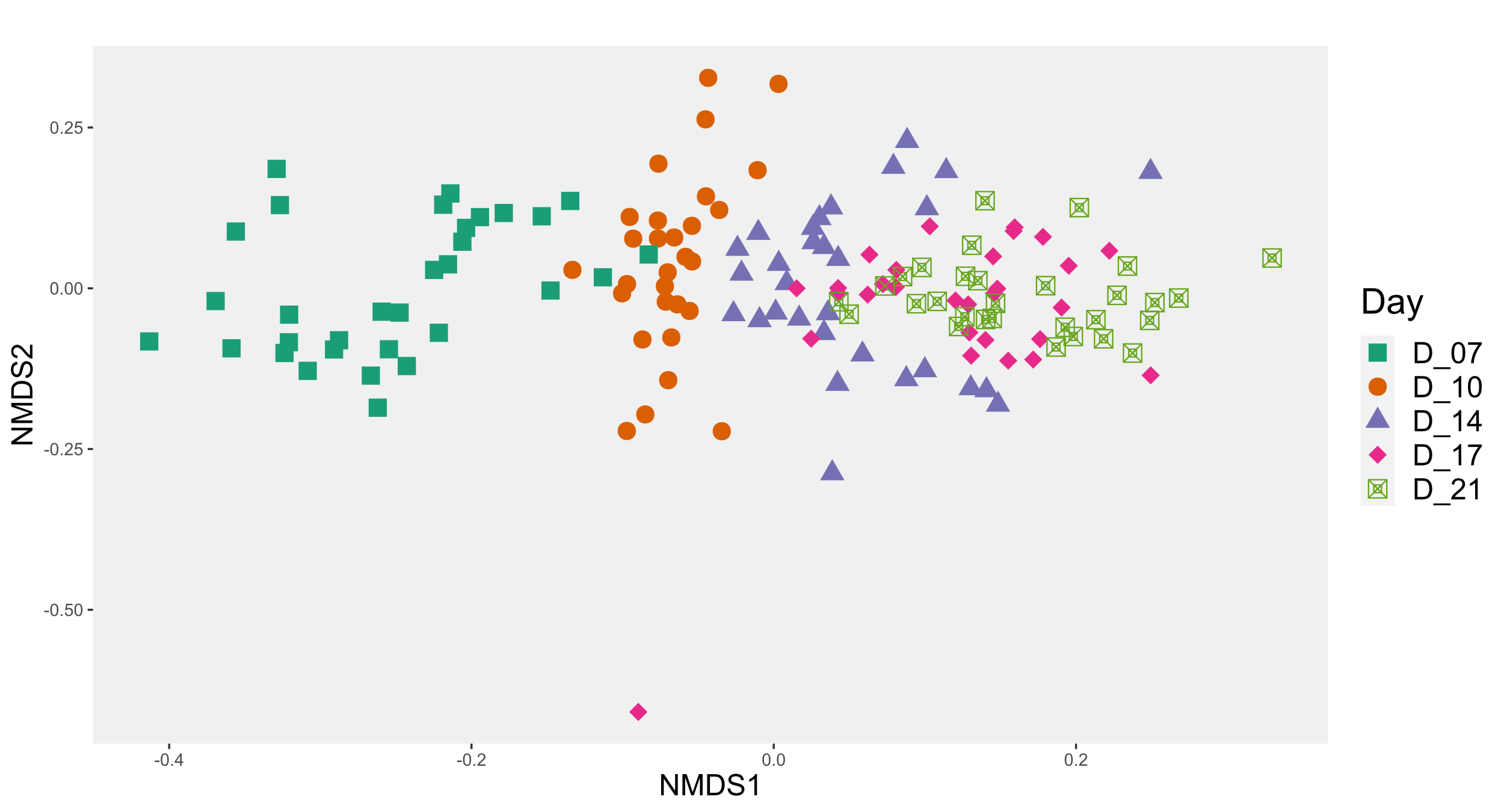


**Figure S3: Graphic** **represented with Non-metric MultiDimensional Scaling (NMDS) of all caecal sequenced samples (Bray-Curtis)**

**Table S1: Bacterial genus variations per dates D7, D10, D14, D17, D21 in ileum (coefficient mean treatment versus control) significant p<0.01**

|  | **Coefficient Mean (Treatment vs control)** | | | | |
| --- | --- | --- | --- | --- | --- |
| **Bacterial genus** | **D07** | **D10** | **D14** | **D17** | **D21** |
| *Bacilli_unclassified.Bacilli_unclassified* |  |  | 0,9 |  |  |
| *Brevibacteriaceae.Brevibacterium* |  |  |  |  | -1,3 |
| *Butyricicoccaceae.Butyricicoccus* | -2,3 | 1,4 | 0,8 |  |  |
| *Clostridiaceae.Clostridium_sensu_stricto_1* | 1,8 |  |  | -2,2 | 1,2 |
| *Corynebacteriaceae.Corynebacterium* | 2,4 |  | 1,6 | 0,5 |  |
| *Eggerthellaceae.Eggerthella* |  | 0,9 | 0,9 | 0,7 | -1,1 |
| *Enterobacteriaceae.Enterobacteriaceae_unclassified* | -1,7 | 1,2 |  |  |  |
| *Enterobacteriaceae.Escherichia.Shigella* |  | 1,0 | 1,8 |  | 1,9 |
| *Enterococcaceae.Enterococcus* |  | 1,4 | 2,6 |  |  |
| *Erysipelatoclostridiaceae.Erysipelatoclostridium* |  | 0,9 | 1,1 |  |  |
| *Erysipelotrichaceae.Erysipelotrichaceae_ge* | -1,3 |  |  |  |  |
| *Lachnospiraceae.Anaerostipes* |  |  | 1,2 | 1,2 |  |
| *Lachnospiraceae.Blautia* |  |  | 1,1 |  | -1,7 |
| *Lachnospiraceae.Lachnospiraceae_unclassified* |  |  | 1,0 | 1,1 | -1,4 |
| *Lactobacillales_unclassified.Lactobacillales_unclassified* |  | 0,6 | 0,9 |  |  |
| *Leuconostocaceae.Weissella* | 0,8 | 2,4 | 2,3 | 0,8 | 1,1 |
| *Microbacteriaceae.Leucobacter* |  |  | 0,8 |  |  |
| *Micrococcaceae.Glutamicibacter* |  |  | 2,1 |  |  |
| *Micrococcaceae.Micrococcaceae_unclassified* |  |  |  | -3,2 | -2,9 |
| *Monoglobaceae.Monoglobus* |  |  |  | 0,9 |  |
| *Moraxellaceae.Acinetobacter* |  | -0,9 |  |  |  |
| *Nocardiopsaceae.Nocardiopsis* |  |  |  |  | -1,4 |
| *Peptostreptococcaceae.Peptostreptococcaceae_unclassified* | |  | -2,8 |  |  |
| *Peptostreptococcaceae.Romboutsia* |  |  | -1,6 |  |  |
| *Planococcaceae.Kurthia* |  |  | 1,4 | -1,6 | -1,6 |
| *Planococcaceae.Planococcaceae_unclassified* |  |  | 1,5 |  | -1,2 |
| *Rhizobiaceae.Ochrobactrum* |  |  | 1,1 |  |  |
| *Rhodobacteraceae.Rhodobacteraceae_unclassified* |  |  | 2,2 |  |  |
| *Staphylococcaceae.Jeotgalicoccus* |  |  |  | 1,4 | 1,0 |
| *Staphylococcaceae.Staphylococcus* | -1,1 | 0,8 | 2,0 |  |  |
| *Streptococcaceae.Lactococcus* | 0,8 |  |  |  |  |
| *Streptococcaceae.Streptococcus* | 2,2 |  | 1,8 |  |  |

Table S2 : Bacterial genus variations per dates D7, D10, D14, D17, D21 in caecum (coefficient mean treatment versus control)

|  | **Coefficient Mean (Treatment vs control)** | | | | |
| --- | --- | --- | --- | --- | --- |
| **Bacterial genus** | **D07** | **D10** | **D14** | **D17** | **D21** |
| *Butyricicoccaceae.Butyricicoccaceae_unclassified* |  |  | 0,6 |  |  |
| *Butyricicoccaceae.Butyricicoccus* | -0,8 |  |  |  |  |
| *Clostridia_UCG.014_fa.Clostridia_UCG.014_ge* |  |  | 4,9 |  |  |
| *Clostridia_unclassified.Clostridia_unclassified* |  |  |  | 0,5 | 0,6 |
| *Clostridia_vadinBB60_group_fa.Clostridia_vadinBB60_group_ge* | 7,0 | 2,4 | 1,5 | 0,8 |  |
| *Clostridiaceae.Clostridium_sensu_stricto_1* | 1,5 |  |  | -2,2 |  |
| *Corynebacteriaceae.Corynebacterium* |  |  |  | 0,8 | 1,0 |
| *Eggerthellaceae.Eggerthella* | 6,3 | 1,7 |  |  |  |
| *Enterobacteriaceae.Enterobacteriaceae_unclassified* | -1,3 | 1,4 | -2,2 |  |  |
| *Enterobacteriaceae.Escherichia.Shigella* |  | -0,6 |  |  |  |
| *Enterobacteriaceae.Salmonella* |  | -0,1 | 2,0 | 4,4 | 4,5 |
| *Enterococcaceae.Enterococcus* |  |  | 0,6 |  |  |
| *Erysipelatoclostridiaceae.Coprobacillus* |  |  | 3,6 | 2,2 | 5,8 |
| *Erysipelatoclostridiaceae.Erysipelatoclostridium* | 1,2 |  | -0,4 | -0,4 |  |
| *Erysipelotrichaceae.Erysipelotrichaceae_ge* |  |  | -0,8 |  | 1,5 |
| *Erysipelotrichaceae.Merdibacter* |  |  | 8,0 |  |  |
| *Lachnospiraceae.Anaerostipes* |  |  |  |  | 0,4 |
| *Lachnospiraceae.ASF356* |  |  | -1,3 | -0,9 | -0,5 |
| *Lachnospiraceae.Eisenbergiella* |  | -7,0 | -1,0 |  | -0,5 |
| *Lachnospiraceae.GCA.900066575* |  | -4,6 | -2,7 | -0,9 |  |
| *Lachnospiraceae.Lachnoclostridium* |  |  | 0,4 | 0,4 | 0,4 |
| *Lachnospiraceae.Lachnospiraceae_unclassified* |  | 0,2 | 0,3 | 0,2 | 0,3 |
| *Lachnospiraceae.Sellimonas* |  |  | -6,0 | -0,9 | -0,9 |
| *Lactobacillaceae.Lactobacillus* |  |  | -0,4 | -0,6 | -0,4 |
| *Lactobacillales_unclassified.Lactobacillales_unclassified* |  |  |  | -0,7 |  |
| *Leuconostocaceae.Weissella* |  | 2,8 | 2,3 | 1,5 | 1,1 |
| *Oscillospiraceae.Colidextribacter* | -1,2 |  | 0,5 |  |  |
| *Oscillospiraceae.Flavonifractor* |  |  |  | 0,6 |  |
| *Oscillospiraceae.Oscillibacter* | -0,4 |  |  |  |  |
| *Oscillospiraceae.Oscillospiraceae_unclassified* | 1,0 | -0,9 | -1,7 | -1,0 |  |
| *Oscillospirales_fa.Oscillospirales_ge* | 7,9 |  |  |  |  |
| *Oscillospirales_unclassified.Oscillospirales_unclassified* |  |  | 2,0 |  | -0,5 |
| *Peptostreptococcaceae.Clostridioides* |  | -1,2 | -0,5 |  |  |
| *Peptostreptococcaceae.Intestinibacter* |  | -1,5 |  |  |  |
| *Peptostreptococcaceae.Peptostreptococcaceae_unclassified* | | 1,8 |  |  |  |
| *Peptostreptococcaceae.Romboutsia* | 0,5 | 0,8 | -1,1 | -0,9 |  |
| *Planococcaceae.Planococcaceae_unclassified* |  |  |  | -2,8 | -1,1 |
| *Ruminococcaceae.Anaerotruncus* | 4,0 | 0,5 | -0,5 | -0,5 | -0,5 |
| *Ruminococcaceae.Caproiciproducens* | 1,5 |  | 0,4 |  |  |
| *Ruminococcaceae.DTU089* |  |  | 0,4 | 0,5 | 0,4 |
| *Ruminococcaceae.Faecalibacterium* |  |  | 4,6 |  |  |
| *Ruminococcaceae.Incertae_Sedis* |  | 0,6 | 0,7 | 0,6 |  |
| *Ruminococcaceae.Negativibacillus* | -1,8 | 0,1 | 0,4 | 0,3 | -0,2 |
| *Ruminococcaceae.Ruminococcaceae_unclassified* | 0,8 | 0,6 | 0,4 | 0,8 | 0,6 |
| *Ruminococcaceae.Subdoligranulum* |  |  |  | 8,4 | 3,2 |
| *Ruminococcaceae.UBA1819* |  |  |  | 0,8 |  |
| *Ruminococcaceae.uncultured* | -1,4 |  |  |  |  |
| *Staphylococcaceae.Jeotgalicoccus* |  |  |  | 1,6 | 1,3 |
| *Staphylococcaceae.Staphylococcus* |  |  | 1,9 |  |  |
